# Editorial: Advances in the study of polyploid evolution in wild populations

**DOI:** 10.3389/fpls.2023.1335981

**Published:** 2024-01-04

**Authors:** Egizia Falistocco, Pilar Prieto, Marilena Ceccarelli, Muhammad Awais Farooq

**Affiliations:** ^1^ University of Perugia, Perugia, Italy; ^2^ Department of Agricultural, Food and Environmental Sciences, University of Perugia, Perugia, Italy; ^3^ Institute for Sustainable Agriculture, Spanish National Research Council (CSIC), Córdoba, Spain; ^4^ Department of Chemistry, Biology and Biotechnology, University of Perugia, Perugia, Italy; ^5^ State Key Laboratory of North China Crop Improvement and Regulation, Agricultural University of Hebei, Baoding, China

**Keywords:** polyploidy, wild populations, evolution, adaptation, polyploid complexes

Polyploidy is one of the most important evolutionary events in plants and a major factor in speciation and plant diversification. About half of all higher plant species are categorized as recent polyploids; in addition, all species in which the genomes have been sequenced, have had in their ancestry at least one polyploidy event, a phenomenon known as whole-genome duplication (WGD) (Alix et al., 2017). The importance of polyploidy in plant evolution is well expressed with the concept “life on earth is predominantly a polyploid phenomenon” (Bennett, 2004) and largely confirmed by research in the past decades. Polyploidy is particularly frequent in wild species and in natural habitats. In this Research Topic “*Advances in the Study of Polyploid Evolution in Wild Populations*” seven articles have been published which cover major themes of this Research Topic: the emergence and establishment of polyploid taxa, cytotype distribution, polyploid complexes, mixed-ploidy populations, and chromosome rearrangement after polyploidization.

The appearance and the success of polyploid lineages is often associated with the ability of polyploid individuals to adapt to various stress conditions and to colonize habitats unsuitable for the diploid ancestors. Thus, the frequency and the geographical distribution of polyploids in given areas should be closely related to the climatic history and ecogeographical heterogeneity of these areas.

The study of Afonso et al. on *Linum suffruticosum* s.l. (Linaceae), a polyploid complex distributed throughout the western Mediterranean basin, revealed that the mosaic distribution of cytotypes is not fully explained by the environmental conditions. Reproductive and competitive interactions among cytotypes could have determined their current diversity and distribution. This study provides relevant information on mechanisms underlying the formation and persistence of polyploids.

*Jasione maritima* (Campanulaceae) offers an interesting case study of the adaptive significance of polyploidy and the spatial distribution of cytotypes. *J. maritima* is a dune species with a parapatric distribution of diploid and tetraploid populations: diploids occurring in the wetter northern region and tetraploids in drier and warmer southern areas of the northwestern Iberian Peninsula (Castro et al., 2020). This pattern suggests that tetraploids were more fit to colonize southern areas than the diploids. In their study Castro et al. assessed the contribution of genome duplications to ecological divergence in the *J. maritima* polyploid complex. They found that WGD had only limited effects on tetraploids that did not result to be more tolerant to water deficit. Castro et al. conclude that further investigations are necessary to clarify the role of ploidy in the distribution patterns of the *J. maritima* complex.

Articles on *Centaurea* (Asteraceae), *Allium* (Amaryllidaceae)*, Menispermum* (Menispermaceae) and *Chrysanthemum* (Asteraceae) demonstrated that organellar genome analysis and genotyping-by-sequencing are excellent tools for resolving systematic controversies and exploring the evolutionary history of polyploid complexes.

*Centaurea tenorei* s.l. is a polyploid complex including *Centaurea lacaitae* (2*n*=4*x*=36), *C. montaltensis* (2*n*=4*x*=36) and *C. tenorei* s.str. (2*n*=2*x*=18). However, the taxonomic distinctiveness of these species is still unclear. De Luca et al. used a panel of SNPs (Single Nucleotide Polymorphisms) obtained via genotyping-by-sequencing to demonstrate that, contrary to what was previously supposed (Peruzzi, 2008), the morphological variability of the group is independent of ploidy level and that *C. tenorei* s.l. is consistently tetraploid with clear evidence of admixture. The three taxa appear to be of hybridogenous origin but some populations have unique genetic features and may be regarded as significant evolutionary units. The study of De Luca et al. confirms SNPs markers as a powerful tool for analyzing the genetic structure of populations characterized by mixed-ploidy, hybridization and introgression.

*Allium* is a large genus exhibiting a great variety of chromosome numbers deriving from polyploidy and dysploidy events. Yang et al. used plastome analysis to explore the complex phylogeny of this genus giving attention to subgenera *Anguinum* and *Rhizirideum*. Within subg. *Anguinum* they identified two strongly supported sublineages in East Asia and Eurasia-America. *Allium tricoccum* exclusively grows in North America but belongs to the Eurasian clade. The distinct taxonomic status of *Allium ulleungense* and its sister taxon were also determined. In subg. *Rhizirideum* the authors explored the evolutionary lineages of some species and estimated the time of their divergence. Data suggest that interspecific hybridization and polyploidization led to the diversification of species in this subgenus during the Pleistocene.

*Menispermum*, a small genus in the large family of Menispermaceae, includes *Menispermum dauricum*, *Menispermum canadense* and *Menispermum mexicanum* distributed in East Asia, Eastern North America and Mexico, respectively. The three species are tetraploid with the chromosome number 2*n*=52. The taxonomic identity of *M*. *mexicanum* is somewhat doubtful. It is considered as a variety of *M. dauricum* or a synonym of *M. canadense* (Calderón de Rzedowski, 1999). Song et al. determined the identity of this uncertain species by using plastome data and nuclear ITS1 and ITS2 sequences. They found that *M. mexicanum* is nested within the clade *M. canadense* demonstrating that *M. mexicanum* is a synonym of *M. canadense*. They also identified important molecular variations in the plastomes of Menispermaceae which could be particularly useful in future studies on the phylogeny and phylogeography of this family.

*Chrysanthemum indicum*, a member of the polyploid complex *C. indicum*, includes diploid and tetraploid cytotypes (Li et al., 2014). Liu et al. in their study, sequenced and assembled the plastomes and mitogenomes of diploid and tetraploid *C. indicum* and used the published plastome and mitogenome sequences of 27 species to explore differences in sequence evolution between the organellar genomes. The organellar genomes of diploid and tetraploid *C. indicum* were similar but the tetraploid *C. indicum* and *C. indicum* var. *aromaticum* contained unique sequences in the mitogenomes. Mitogenome structure varied greatly across *Chrysanthemum*, whereas the plastome is structurally conserved across Asteraceae. These results are important for studying *Chrysanthemum* organellar genome evolution with possible applications to conservation and breeding.

Genomic changes and chromosome rearrangements following the polyploidization process have been extensively investigated during the last decades. Tomaszewska et al. aimed at showing genome structural diversity and genome relationships in tetraploid, hexaploid and octoploid *Avena* species, including amphiploids, and determining patterns of intergenomic translocations across different oat taxa. The FISH results showed that the A, B, C, and D genomes of oats differ significantly in their involvement in non-centromeric, intercalary translocations and distal intergenomic translocations.There was a predominance of distal intergenomic translocations from the C- into the D-genome chromosomes. Translocations from A- to C-, or D- to C-genome chromosomes were less frequent, proving that at least some of the translocations in oat polyploids are non-reciprocal. Translocations and other rearrangements may be useful in breeding through introgression from wild species because they contain desirable gene combinations, but they could also be an obstacle because they restrict recombination.

Overall, the articles published in this Research Topic are excellent examples of recent advances in our knowledge of wild polyploids. The studies comprise very different taxa and have been carried out with the use of state-of-the-art techniques. We thank all the authors for their contributions and the Editorial Office for having provided us support for the realization of this Research Topic “*Advances in the Study of Polyploid Evolution in Wild Populations*”.

## Author contributions

EF realized the Editorial. MC, PP and AF revised and approved the submitted version.
